# Crosstalk between Glycogen-Selective Autophagy, Autophagy and Apoptosis as a Road towards Modifier Gene Discovery and New Therapeutic Strategies for Glycogen Storage Diseases

**DOI:** 10.3390/life12091396

**Published:** 2022-09-08

**Authors:** Marina Andjelkovic, Anita Skakic, Milena Ugrin, Vesna Spasovski, Kristel Klaassen, Sonja Pavlovic, Maja Stojiljkovic

**Affiliations:** Institute of Molecular Genetics and Genetic Engineering, University of Belgrade, Vojvode Stepe 444a, 11010 Belgrade, Serbia

**Keywords:** glycogen storage diseases, glycogen-selective autophagy, autophagy, apoptosis, modifier genes, autophagy-inducing drugs

## Abstract

Glycogen storage diseases (GSDs) are rare metabolic monogenic disorders characterized by an excessive accumulation of glycogen in the cell. However, monogenic disorders are not simple regarding genotype–phenotype correlation. Genes outside the major disease-causing locus could have modulatory effect on GSDs, and thus explain the genotype–phenotype inconsistencies observed in these patients. Nowadays, when the sequencing of all clinically relevant genes, whole human exomes, and even whole human genomes is fast, easily available and affordable, we have a scientific obligation to holistically analyze data and draw smarter connections between genotype and phenotype. Recently, the importance of glycogen-selective autophagy for the pathophysiology of disorders of glycogen metabolism have been described. Therefore, in this manuscript, we review the potential role of genes involved in glycogen-selective autophagy as modifiers of GSDs. Given the small number of genes associated with glycogen-selective autophagy, we also include genes, transcription factors, and non-coding RNAs involved in autophagy. A cross-link with apoptosis is addressed. All these genes could be analyzed in GSD patients with unusual discrepancies between genotype and phenotype in order to discover genetic variants potentially modifying their phenotype. The discovery of modifier genes related to glycogen-selective autophagy and autophagy will start a new chapter in understanding of GSDs and enable the usage of autophagy-inducing drugs for the treatment of this group of rare-disease patients.

## 1. Introduction

Glycogen is a key source of energy in the body during fasting, and its metabolism is central in the maintenance of an optimal level of glucose in the blood. A complex system of different proteins is responsible for glycogen metabolism, including enzymes performing synthesis, branching, debranching, degradation, etc. [1]. Twenty-three different monogenic disorders, glycogen storage diseases (GSD 0a, 0b, Ia, Ib, II, IIb, IIIa, IIIb, IIIc, IV, V, VI, VII, IXa, IXb, IXc, IXd, X, XI, XII, XIII, XIV and XV), and inborn errors of metabolism are due to defects in genes encoding proteins involved in the metabolism of glycogen (*GYS2*, *GYS1*, *G6PC*, *SLC37A4*, *GAA*, *LAMP2*, *AGL*, *GBE*, *PYGM*, *PYGL*, *PFKM*, *PHKA2*, *PHKB*, *PHKG2*, *PHKA1*, *PGAM2*, *LDHA*, *ALDOA*, *ENO1*, *PGM1* and *GYG1*). In addition to these diseases, there are a few “secondary glycogenoses”, also monogenic diseases, that are caused by defects in proteins indirectly involved in glycogen processing (*PRKAG2*, *EPM2A* and *NHLRC1*) [2]. The combined incidence of glycogen storage diseases in the population is estimated at 1 case per 20,000–43,000, putting them in the group of rare diseases [3]. Except GSD II (Pompe disease), for which an enzyme-replacement therapy and antibody–enzyme fusion therapy exist, other glycogen storage diseases are currently incurable [4,5]. Glycogen storage diseases are very complex disorders with many systems involved. Clinically, glycogen storage diseases affect the liver, muscles, or both. In this review, glycogen storage diseases will be represented by hepatic glycogen storage disease types Ia and Ib. The phenotype of GSD Ia is characterized by the abnormal storage of glycogen in the liver (hepatomegaly) and kidneys, hypoglycaemia, short stature, hepatic adenomas, renal calcification, and chronic kidney disease. In addition to these symptoms, patients with GSD Ib usually also develop neutropenia, inflammatory bowel disease, and iron-resistant anemia [6,7]. In the majority of world population, GSD Ia accounts for approximately 80% of GSD I cases, but exceptions have also been documented [8]. The phenotypic spectra of both GSD Ia and GSD Ib are very wide, making it impossible to accurately predict disease onset and disease course based solely on the variants identified in the disease-causing genes (*G6PC* and *SLC37A4*, respectively). Lack of correlation between individual mutations and the presence of neutropenia, bacterial infections and systemic complications in patients with GSD Ib have been reported [9]. Therefore, Melis and co-workers suggested that different modifier genes may modulate neutrophil differentiation, maturation, and apoptosis, and thus influence the severity and frequency of infections [9]. Additionally, recent findings of Saban and co-workers have suggested that whole-exome sequencing should be considered in GSD Ia patients who show persistent symptoms despite optimal dietary management, as they were able to find variants in the gene for sucrase isomaltase to explain patients’ conditions [10].

Indeed, decades of research on monogenic diseases have firmly established the statement that although a defect in only one gene is enough for disease to fully develop, the correlation between genotype and phenotype in monogenic disorders is not simple at all [11]. Ideally, if we knew all the genetic pieces of the puzzle, and all genetic modifiers, we would be able to accurately predict variant clinical manifestations, which would be of great value for the prognosis and treatment of persons with genetic diseases. In general, the stability of a protein is a result of the systems that tend to produce it and protect it (synthesis pathways, absorption pathways, intracellular molecular chaperones, etc.), or degrade it and eliminate it (proteolytic systems, excretion pathways, etc.). Genetic modifiers can increase or decrease the severity of a certain disease, but may not be disease-causing themselves [12]. The role of genetic modifiers in different rare diseases, such as left ventricular noncompaction or fragile X syndrome, is well documented [13,14]. It has also been shown that variants within the *KLF1* gene could ameliorate the severity of beta-thalassemia syndromes by indirectly facilitating the production of gamma-globin polypeptide chains that can take over the function of defective beta-globin chains [15,16]. This makes KLF1 a genetic modifier of beta thalassemia syndromes, and a very potent target for gene therapy for these disorders.

Having in mind the complexity of metabolic pathways and the number of enzymes that are included along the way, there is a need to address modifier genes relevant for disorders of glycogen storage metabolism. A recent metabolomics study found that the metabolic defect of GSD Ia and GSD Ib has profound effects on a variety of metabolic pathways, in addition to the known typical abnormalities [17]. Thus, modifier genes may be found in any of the affected pathways. However, since the majority of glycogen storage diseases share the etiology of excessive amounts of normal or defectively branched glycogen, glycogen is an obvious starting point in the quest for modifier genes. The hypothesis to search for modifiers among genes related to autophagy comes from the recentstudy that emphasized the importance of autophagy for disorders of glycogen metabolism [18]. A selective autophagy pathway trafficking glycogen to the lysosome has been established as a non-canonical pathway of glycogenolysis [19].

Therefore, in this manuscript, we identify the genes involved in glycogen-selective autophagy as potential modifiers of glycogen storage disorders. Given the small number of genes associated with glycogen-selective autophagy, we also include genes, transcription factors and non-coding RNAs involved in autophagy. A cross-link with apoptosis is also addressed. All of these genes could be analyzed in GSD patients with unusual discrepancies between genotype and phenotype in order to discover genetic variants potentially modifying their phenotype. Discovering new modifier genes related to glycogen-selective autophagy or autophagy could lead to new therapeutic options for this group of rare-disease patients.

## 2. Glycogen-Selective Autophagy

The highly conserved catabolic process of cell-component degradation that promotes the recycling of essential cellular components and enables the preservation of intracellular homeostasis is autophagy [20,21]. During nutrient deficiency, non-essential proteins are degraded in the lysosome, and amino acids are recycled and used for gluconeogenesis, ATP production, or the synthesis of new proteins [22]. Additionally, large glycogen polymers can be broken down and used as a source of free glucose for glycolysis [18]. This type of autophagy process that is more selective and requires the coordination of several protein complexes and vesicle fusion events for organelle and macromolecule degradation is called glycogen-selective autophagy [21]. Glycogen is a multibranched polysaccharide of glucose that serves as a form of energy storage; thus, glycogen-selective autophagy has a role in cell glycogen degradation and promotes the maintenance of glucose homeostasis under the condition of high glucose demand [23]. In the same way as glycogen from lysosome, cytosolic glycogen is degraded by glycogen phosphorylase into phosphorylated glucose (glucose-1-phosphate) for glycolytic metabolism [19].

### 2.1. Signal Pathways Involved in Glycogen-Selective Autophagy

Glycogen-selective autophagy is mainly induced by the cyclic AMP (cAMP)/protein kinase A signal pathway. In response to the extensive demand for glucose production, the formation of autophagic vacuoles and the degradation of glycogen inside these organelles are stimulated and increased by cAMP [24,25]. Moreover, chemical agents that stimulate the production of cAMP also stimulate glycogen-selective autophagy in the liver, heart, and skeletal muscles, which indicates that the cAMP/protein kinase A signal pathway has a key role in this process [26,27]. In lysosomes, glycogen-selective autophagy is activated by calcium, and it is known from the literature that cAMP can modify the activity of the calcium pump and open calcium channels [28,29] through the activity of the membrane-bound Ca^2+^-ATPase, thus increasing the entry of calcium into lysosomes [30]. Calcium level within lysosomes influences the activation of glycogen-hydrolyzing acid alpha-glucosidase and contributes to the existence of glucose in a non-phosphorylated state, as well as facilitates the transport of glucose from the lysosomal membrane to the cytoplasm [31,32]. The third pathway involved in glycogen-selective autophagy is the mTOR complex 1 signal pathway which has the ability to reduce glycogen-selective autophagy by regulating the activity of protein phosphatase 2A (PP2A) [33,34]. PP2A is regarded as the target of cAMP, glucagon, and insulin, and has a role in the stimulation of acid alpha-glucosidase synthesis, while the activation of mTOR reduces glycogen-selective autophagy by inhibiting PP2A [35,36]. Mammalian target of rapamycin complex 1 (mTORC1) negatively regulates glycogen-selective autophagy by phosphorylating ULK1 [37] and autophagy/beclin-1 regulator 1 in order to ubiquitinate ULK1 for degradation [38]. Conversely, during amino acid starvation, which induces autophagy, the mTORC1 signal pathway is inhibited [39]. Hepatic DEP domain-containing mTOR-interacting protein accelerates the inhibition of mTORC1 during the transition to fasting to adjust metabolism to the nutritional status [40].

Altogether, cAMP, calcium, and the mTOR signaling pathways are involved in the regulation mechanism of glycogen-selective autophagy, indicating that this is a highly regulated process [21].

### 2.2. Glycogen-Selective Autophagy and Glycogen Storage Disease I

Given that glycogen-selective autophagy is a very important process in metabolism homeostasis, and that glycogen mishandling is centrally involved in the pathophysiology of different metabolic diseases in a wide range of tissues, a hypothesis has been set that the restoration of autophagy might improve outcomes in glycogen storage diseases in which glycogen-selective autophagy is known to be defective [18,19].

GSD Ia is caused by pathogenic genetic variants in *G6PC*, a gene for glucose-6-phosphatase, while GSD Ib is caused by variants in *SLC37A4* (G6PT), a gene for glucose-6-phosphate transporter [41]. SLC37A4 transports glucose-6-phosphate from the cytoplasm to the lumen of the endoplasmic reticulum by complexing with G6P (in the liver, kidney, and intestine) or -P (in neutrophils) during gluconeogenesis or glycogenolysis to maintain glucose homeostasis. Interestingly, recent studies have shown that SLC37A4 has the ability to negatively regulate mTOR complex 1, which functions as an inhibitor of glycogen-selective autophagy [42]. Hye-Hyun Ahn and co-workers performed a screening of potential activators of autophagy upstream of ULK1, which is required for the initiation step of autophagy. Among 500 genes that encode ER or lysosome proteins, *SLC37A4* was the most potent activator of the autophagy function. The overexpression of *SLC37A4* seems to promote the initial step of glycogen-selective autophagy acting upstream of mTORC1 by increasing the interaction between *ULK1* and *Atg9* (autophagy-related gene 9), improving autophagic flux independent of SLC37A4 transport activity [42]. Furthermore, to assess the effects of SLC37A4 on autophagy, they generated a stable *SLC37A4* knockdown of Hep3B cells and measured levels of LC3-II, ubiquitin conjugates, and an autophagy substrate p62. In comparison with wild-type cells, Hep3B/SLC37A4 knockdown cells exhibited a reduced basal level of LC3-II and an increased accumulation of ubiquitin-conjugates. In the same way, the level of endogenous p62 aggregates was enhanced in SLC37A4-knockdown cells. Consistent with the stimulatory role of *SLC37A4* expression on autophagy, these results indicate that SLC37A4 is crucial in autophagy activation [42]. Furthermore, experiments performed on generated transport activity-dead mutants of SLC37A4 indicate that the SLC37A4 autophagy-stimulatory activity of SLC37A4 is independent of its transport activity, and that its role in autophagy may be one of the additional functions of SLC37A4 [42]. In light of this recent finding, it would be very interesting to explore the effect of deficient SLC37A4 transporter on the process of glycogen-selective autophagy in a model system for glycogen storage disease Ib (e.g., in a patient-specific iPSC-derived hepatocyte-like cellular model system).

### 2.3. Genes Involved in Glycogen-Selective Autophagy

**Figure 1:**
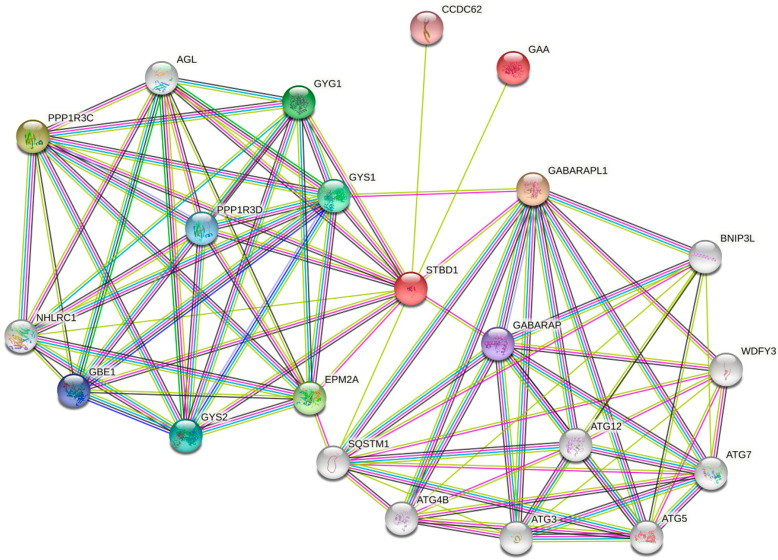
Protein–protein interaction network between STBD1, GABARAPL1 and GAA has been developed using STRING, a database of known and predicted protein–protein interactions.

The current state of evidence suggests that glycogen-selective autophagy protein machinery involves starch-binding domain-containing protein 1 (STBD1) as the receptor [43], GABA type A receptor-associated protein-like 1 (GABARAPL1) as its Atg8 (autophagy-related genes) partner [44], and acid α-glucosidase (GAA) as the lysosomal glycogen degradation enzyme. The interconnection between these three proteins and functional network in which they belong are represented in the Figure 1. Thus, additional protein mediators may emerge in future research studies.

Amino acid sequence-based computational predictions and colocalization studies identified STBD1 as the primary glycogen-selective autophagy receptor, as it contains both a glycogen-binding domain (CBM20) and several Atg8-interacting motifs (AIMs) [45]. The role of STBD1 is to recruit glycogen into the autophagosome (in the literature, known as glycophagosome) by binding to GABARAPL1 [44]. In vitro co-expression studies have revealed that STBD1 interacts with all Atg8 protein family members, with the highest binding affinity for GABARAPL1 [43]. The mature glycophagosome fuses with a lysosome where GAA degrades glycogen to free glucose for metabolic recycling. It is unknown how glucose is released from the phagolysosome following glycogen degradation. Some reports show that the glucose transporter Glut8 and the transmembrane protein spinster 1 (Spns1) may transport glucose out of the lysosome [46,47], but future studies are required for validation.

The glycophagosome is formed by binding the Atg8 protein partner to the STBD1–glycogen complex. The mammalian Atg8 protein family consists of two subfamilies, the Lc3 (Lc3a, Lc3b, Lc3c) and Gabarap (GABARAP, GABARAPL1, GABARAPL2) [45,48,49,50]. Therefore, in addition to STBD1, other proteins were identified by computational analysis to contain AIMs in their amino acid sequence: glycogen synthase (GYS1), glycogen branching enzyme (GBE1), glycogen phosphorylase (PYGM, PYGL, PYGB), glycogenin (GYG1, GYG2), glycogen debranching enzyme (AGL), laforin (EMP2A), and malin (NHLRC1) [19]. Since the presence of an AIM in a glycogen-binding protein does not necessarily translate to functional binding with Atg8 proteins, further work is required to fully elucidate their function as potential glycogen-selective autophagy receptors.

### 2.4. Other Genes Involved in Autophagy

The transition of autophagy studies from morphology to molecular machinery relies on the identification of *Atg* in yeast [48]. To date, approximately 30 *Atgs* have been reported to be required for autophagosome biogenesis. Among these genes, one subgroup containing approximately 18 genes—shared among the various types of nonselective and selective autophagy and required for autophagosome formation—is termed core autophagy machinery [49]. The core Atg proteins can be divided into four functional subgroups: (i) the Atg1/ULK complex which participates in the regulation of autophagosome formation; (ii) Atg9 and its cycling system (Atg2, Atg9, and Atg18) which have a role in membrane delivery; (iii) the PtdIns 3-kinase (PtdIns3K) complex which has a role in vesicle nucleation and in the recruitment of PtdIns3P-binding proteins; and (iv) two Ubl (ubiquitin-like) conjugation systems, Atg12 and Atg8, which play roles in vesicle expansion [50,51,52].

In mammalian cells, ULK1 (Unc-51-like autophagy activating kinase 1), a serine/threonine kinase, is one of the core Atg proteins required for the initiation step of autophagy, as well as glycogen-selective autophagy. ULK1 forms complexes with other Atgs [53,54] and the complexes are mostly controlled by the mTOR complex 1 (mammalian target of rapamycin complex 1) [37,54]. Among other Atg proteins, Atg9 is a membrane protein containing six transmembrane domains functionating as a shuttle between autophagosomes and the trans-Golgi network during the biogenesis of autophagosomes [55]. The movement of Atg9 is dependent on the activity of the ULK1 that regulates autophagosome biogenesis by delivering membrane sources derived from the trans-Golgi network. Atg9 ATG9 seems to have a conserved role in coordinating membrane transport and displays a cycling pattern [37,56].

There are two types of core Atg proteins involved in vesicle nucleation and in the recruitment of the PtdIns3P-binding proteins known as complex I (PtdIns3K-C1) and complex II (PtdIns3K-C2). PtdIns3K-C1 has a crucial role in autophagy initiation, while PtdIns3K-C2 functions in autophagosome maturation, endocytosis, and Atg8/LC3-associated phagocytosis (LAP) [57,58]. 

## 3. Cross-Talk between Autophagy and Apoptosis

In response to various stimuli, the destiny of the cell is determined by multiple signaling pathways. Autophagy and apoptosis are two distinct pathways that largely interact, thus playing a crucial role in the life and death of a cell. Autophagy is important for cell survival in times of starvation, or as a cell cleaner under certain types of stress, while apoptosis is a programmed cell death. 

To preserve normal physiology and tissue function, cells that have an aberrant function, some damage, or are no longer necessary, are constantly cleared through apoptosis, which is executed by two well-characterized pathways: the intrinsic and the extrinsic apoptosis pathways [59,60,61]. Apoptosis is classified into type I, type II, and type III programmed cell death. Type I is classic apoptosis, also known as caspase-dependent apoptosis; types II and III are caspase-independent apoptosis. Type II is characterized by the appearance of the autophagic and double membrane of the vacuole, while type III occurs without the condensate chromatin and has not yet been sufficiently examined [62].

Recently, autophagy has also been linked to the process of cell’s self-elimination [63]. The cross-talk between autophagy and apoptosis can be three-fold [64]. The autophagy pathway can act as a partner, antagonist or enabler of apoptosis. In the first-case scenario, both pathways are rushing to lead the cell to its extinction. In the second case, autophagy recycles nutrients or removes damaged organelles/protein aggregates, etc., in order to prolong the cell’s life, and this negatively regulates apoptosis. In the third case, autophagy is a prologue to apoptosis: it enables enough energy for the cell to undergo apoptosis. Therefore, it is not a surprise that numerous genes are shared by both pathways (such as *P53, BCL2, ATG5* and *P14ARF*). 

Autophagy and apoptosis may be triggered by common upstream signals, and on a molecular level, this means that these two processes share common pathways that either link or separate the cellular responses. Cells can undergo apoptosis or autophagy as a response to the same type of stressors. Selection between apoptosis and autophagy relies on the intensity of the stimulus. Numerous stress mediators such as the elevation of cytosolic Ca^2+^, isoforms of p19ARF (or p14ARF in humans), p53, BH3-only proteins (some members of the BCL-2 protein family), and DAPK family members (death-associated protein kinases) can stimulate both apoptosis and autophagy. Several proteins that have a crucial role in autophagy, such as ATG5, can be cleaved by calpain cysteine proteases, which leads to the loss of pro-autophagy effects, and becoming a pro-apoptotic molecule [65]. 

Autophagy-related genes and those shared between autophagy and apoptosis could carry genetic variants that may modify the phenotypes of human disorders in which these two pathways play an important role. 

## 4. Road towards Modifier Gene Discovery

Taking into account the described mechanisms of glycogen-selective autophagy, autophagy, and the crosstalk with apoptosis, we suggest that patients with a confirmed defect in one of the genes causing glycogen storage diseases that present with an unusual phenotype could be additionally analyzed for the presence of modifier genes. Nowadays, when the sequencing of all clinically relevant genes, whole human exomes, and even whole human genomes is fast, easily available and affordable, we have a scientific obligation to holistically analyze data and draw smarter connections between genotype and phenotype. 

**Figure 2:**
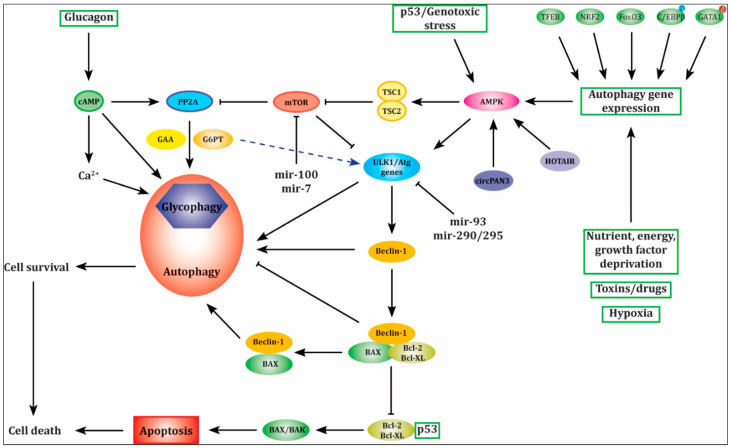
Relation between genes involved in glycogen-selective autophagy and autophagy, genes shared with apoptotic pathway, and genes encoding regulatory factors, such as transcription factors and non-coding RNA (miRNAs, lncRNA and circRNA) as a complex network for the discovery of modifier genes and new therapeutic strategies for glycogen storage diseases.

Only a few genes have been described as a part of glycogen-selective autophagy protein machinery. Therefore, we suggest going beyond genes related to glycogen-selective autophagy and analyze genes related to autophagy as well—an extensive list of genes is given in a review by Bordi and co-workers [20]. It should be noted that each variant found in genes shared between autophagy and apoptosis (*P53, BCL2, ATG5* and *P14ARF*) should be evaluated with caution as it could exert dual effect. Furthermore, we list genes encoding regulatory factors related to autophagy, such as transcription factors and non-coding RNA (Table 1) [66,67,68,69,70,71,72,73,74,75]. In addition, the interrelation between players of this complex network is depicted in Figure 2.

A recent study of Klaassen and co-workers described the *SHANK* gene family as a candidate modifier for a rare metabolic disease—phenylketonuria [76]. The whole-genome sequencing of patients with markedly unusual genotype–phenotype relations (persons without intellectual disability, although they have genetic defect and have never been treated) pointed to several variants that do not abolish the function of SHANK proteins (apparently benign variants). However, the predicted changes in post-translational modifications of SHANK proteins due to these variants could influence the functioning of the glutamatergic synapses and cytoskeleton regulation, and thus contribute to maintaining the optimal synaptic density and number of dendritic spines. Consequently, these rare variants could modify the phenotype of their carriers. 

An intrinsic problem related to research on rare diseases is low sample sizes insufficient to reach adequate power for studies targeting modifier genes. This issue can be solved by extracting data from collaborative databases and large datasets of patients with rare diseases, such as those stored in the Genome-Phenome Analysis Platform RD-Connect platform (https://rd-connect.eu/what-we-do/omics/gpap/). However, before a larger number of samples is collected, emphasis should be made on the importance of assessing the effect of each variant found in a selected group of genes. Prediction of the effect of variants found in coding gene regions on the protein function can be performed using numerous existing computation tools and algorithms, such as CADD, GERP, Mutation Assessor, MutPred2, PolyPhen-2 REVEL, SIFT, etc. [77,78,79,80]. In addition to individual tools, a useful overview of scores calculated by different tools can be seen when using the Ensemble (http://www.ensembl.org/) and VarSome (https://varsome.com/) databases.

In addition, an in silico three-dimensional model of a protein carrying amino acid substitution or small insertion/deletion can be made with the AlphaFold algorithm, Phyre2 web portal, or Swiss-Pdb Viewer [81,82,83]. These tools require inserting the code of a specific protein from a protein database in order to use a theoretical protein model (preferably human). Some of these tools need to use the Plymel Molecular Graphics System, Schrödinger, LLC, for the visualization of three-dimensional models [76].

Special attention should be paid to rare variants predicted to be pathogenic, as they are expected to change the structure and/or function of a protein. If such a variant resides in a gene described to cause a monogenic disease (e.g., in https://www.omim.org, https://www.orpha.net, https://www.malacards.org, etc.), the phenotype of the patient must be re-evaluated for symptoms of that disease.

### 4.1. Rare vs. Common Variants

Strictly following the rules for the classification of genetic variants [84], only rare variants, found with <0.01 frequency in large databases of healthy persons, such as gnomAD exomes and gnomAD genomes (https://gnomad.broadinstitute.org/), can be considered to be pathogenic. However, in the case of modifier genes, common variants computationally predicted to change the structure/function of the protein involved in the autophagy pathway should also be taken into consideration for their modifier effect. 

An example of this approach was described for late-onset Alzheimer’s disease, where a common variant rs3796529 (MAF > 15%) was described to be a protective variant for hippocampal atrophy [85].

Furthermore, as in multifactorial diseases [86], we can expect that the cumulative effect of the main Mendelian disease-causing locus (major size effect), together with one or more modifier loci carrying rare or common variants (each with a minor size effect), work together to shape what we observe as a disease phenotype of a “monogenic” disease.

### 4.2. Regulatory Role of Non-Coding Regions

In addition to variants in coding regions that might affect the structure and/or function of a protein, non-coding regions of genes related to autophagy also have immense potential to act as modifiers.

#### 4.2.1. Transcription Factors

Microphthalmia/transcription factor E or MiT/TFE family members (MITF, TFEB, TFE3, and TFEC), nuclear factor erythroid-derived 2-like 2 (NFE2L2/NRF2), the forkhead box o (FoxO) family, the CCAAT/enhancer-binding protein (C/EBP) family, and the GATA transcription factor are the most recognized master genes of autophagy (for a comprehensive review, see Kim et al., 2020) [72].

Therefore, both variants in genes encoding for transcription factors as well as the promoter regions of genes regulated by them are worthy of investigation [73,74,75].

#### 4.2.2. Non-Coding RNA

ncRNAs are functional RNA molecules that regulate gene expression at the transcriptional and post-transcriptional level. Once considered “transcriptional noise”, non-coding RNAs have emerged as potential biomarkers, as well as therapeutic targets in many diseases [87,88]. The ncRNA, usually ranging from 20 to 10,000 nucleotides, can modulate physiological responses through different mechanisms, such as RNA–RNA or RNA–protein interactions, and some of the interactions can be stabilized by the different chaperones [89]. Based on the linearity of transcripts, ncRNAs can be classified as linear (microRNAs–miRNAs and long non-coding RNAs–lncRNAs) or circular (circular RNAs–circRNAs).

miRNAs, spanning between 18 and 24 nucleotides, regulate gene expression through base-pairing with complementary sequences of the 3′untranslated region (UTR) of messenger RNAs (mRNA). This interaction results in gene silencing accomplished through a combination of translational repression and mRNA destabilization through its polyA tail shortening or inhibiting the mRNA translation into proteins [90].

Recently, miRNAs have emerged as new players in the processes of autophagy regulation. Namely, it has been shown that different miRNAs target mRNAs of several key proteins involved in different stages of autophagy. The mTOR gene, involved in the induction stages of autophagy, has been described as the target of several miRNAs, including miR-100, which has been shown to directly suppress the expression of mTOR, thus inducing autophagy in hepatocellular carcinoma cells (HCC) [67]. Similarly, it has been demonstrated that mTOR, a negative regulator of autophagy, is a direct target of miR-7, whose overexpression in HCC leads to increased autophagy [68]. Almost all core autophagy pathway elements are subject to miRNA-dependent fine-tuning. For instance, it has been shown that targeting ULK1 by miR-290/295 results in the suppression of autophagic activity in response to glucose starvation [69]. Additionally, it has been reported that miR-93 targets ULK1 and reduces its protein levels under hypoxia conditions, leading to the inhibition of hypoxia-induced autophagy. The re-expression of ULK1 without miR-93 response elements restores this miR-93-suppressed autophagy [70].

The number of miRNAs involved in the regulation of autophagy-related genes is constantly increasing and the understanding of this process is further complicated by the fact that several key autophagy genes are being controlled by the same miRNA. Namely, it has been reported in several independent studies, that previously mentioned miR-7 is also involved in the regulation of LKB1, ULK2, ATG4A and ATG7 autophagy-related targets [71].

It has also been shown that by the deregulation of miRNA expression, autophagic activity can be triggered by a number of stress-inducing stimuli such as nutrient, energy and/or growth factor deprivation, hypoxia, different toxins, and drugs. 

Another level of complexity of miRNA-regulated autophagy arises from the findings that some MREs, and even miRNAs, show genetic variants in different individuals. In CLL patients, for example, variants were detected in two autophagy-related miRNAs, miR-16 and miR-29B, but the effect of these variants on autophagy was not analyzed [91,92]. To the best of our knowledge, no study directly has assessed the effect of miRNA/MRE variants on the autophagic responses of cells or organisms to date.

Recent advances in research technologies have made miRNAs attractive tools in disease diagnosis, treatment monitoring, and follow-up. On the other hand, autophagy modulation has recently been considered in the treatment of a spectrum of diseases, which is why a comprehensive knowledge of miRNAs and their involvement in autophagy regulation might contribute to the research on autophagy modulation as an innovative treatment approach for a number of diseases [93].

lncRNAs encompass a large and highly heterogeneous group of ncRNA transcripts longer than 200 nucleotides that differ in their biogenesis and genomic origin. Although the functionality of most of the estimated 100,000 human lncRNAs is not clear, a growing number of lncRNAs do have an important cellular function, including autophagy [94,95]. A unique feature of lncRNAs is their biochemical ability to interact with a variety of molecules, such as RNA, DNA, and proteins, forming—through lncRNAs’ specific RNA functional domains—a number of different complexes.

LncRNAs generally modulate autophagy via regulating the expression of different ATG genes. For example, lncRNA HOX transcript antisense RNA (HOTAIR) can enhance autophagy through the regulation of ATG3 and ATG7 in HCC [96]. It has also been shown that this lncRNA can be involved in the pathogenesis of the disease through the enhancement of autophagy by modulating the AMPK/mTOR/ULK1 pathway [97]. lncRNA DCST1-AS1, as a carcinogenic factor, has been shown to be closely related to the autophagosome formation and the regulation of autophagy-related genes in HepG2 cells and inhibit autophagy and apoptosis by modulating the AKT/mTOR signaling cascade. In contrast, the reduction in expression of this lncRNA hastened apoptosis and stimulated autophagy. The results of this study support the view that the downregulation of lncRNA DCST1-AS1 expression may inhibit HCC progression by accelerating the process of autophagy [98]. Some lncRNAs modulate the process of apoptosis in more than one way. Namely, the expression of lncRNA PTENP1 in HCC suppresses the AKT/mTOR signaling pathway, thus inducing autophagy. Additionally, it has been shown that this lncRNA antagonizes miR-17 and miR-20a, negative regulators of the core autophagy genes *UKL1*, *ATG7* and *p62*, and thus, indirectly induces autophagy by upregulating these autophagy-related genes [99]. 

circRNAs comprise a large class of non-coding RNAs that are produced by a specific type of splicing called backsplicing, during which the downstream splice donor site is covalently linked to an upstream splice acceptor site. The lack of a 5′ cap and 3′ tail make the circular molecules more resistant to RNase degradation compared to their linear equivalent. Most of the circRNAs identified to date have been shown to act as a miRNA sponge and regulate target gene expression by inhibiting miRNA activity. One circRNA can regulate one or multiple miRNAs through multiple miRNA binding sites in the circular sequence [100,101].

circRNAs modulate autophagy by regulating the key molecules involved in the process, thus affecting the biological functions of cells. It has been demonstrated that circPAN3 promotes the phosphorylation and activation of AMPK and inhibits mTOR phosphorylation to promote autophagy. This circRNA also upregulates the expression of *BECN1* and *LC3-II*, thereby further promoting autophagy [102]. Other circRNAs have been shown to activate the PI3K-AKT–mTORC1 signaling pathway by interacting with Mir145-3p, thus inhibiting autophagy. However, it has been demonstrated that cell stimulation with a high glucose concentration leads to the downregulated expression of this autophagy-related circRNA (ARC), thus promoting autophagy [103].

## 5. Therapeutic Implications

Modifier gene discovery is extremely important, not only to duly explain the correlation between genotype and phenotype, but also to point out to new therapeutic strategies. Targeting modifier genes may alleviate symptoms of a disease. In a group of diseases, such as in a group of glycogen storage diseases where similar processes, such as glycogen-selective autophagy, autophagy and apoptosis, are disturbed, the development of one drug targeting a protein involved in autophagy and apoptosis, rather than on disease-causing deficient protein, may become a successful approach.

In some liver diseases where impaired autophagy has been recorded, the therapeutic boosting of hepatic autophagy in animal models has been shown to protect against drug-induced liver injury, alcohol-associated liver disease, and nonalcoholic fatty liver disease [104]. Autophagy-targeting strategies are also established in various human diseases, in cancer and vascular diseases, in viral infections and microbiome-mediated diseases, in proteinopathies (e.g., amyloidopathies, tauopathies, synucleinopathies, and prion diseases), and lysosomal storage diseases [72]. In relation to proteinopathies, for which recent studies have shown that inducing autophagy significantly improves phenotype by directly degrading abnormal protein aggregates [105], the pharmacological modulation of autophagy should be considered as a promising strategy against excessive glycogen in glycogen storage diseases.

The use of autophagy-inducing drugs must be considered with extreme caution because of the crosstalk with apoptotic pathways and the possibility to yield destructive outcomes. Therefore, we must focus our further research on the discovery of modifier genes—the minor players that can be targeted without disrupting the main processes of cellular survival and death. Additionally, in the future, it will be important to understand all processes and signaling pathways that modulate autophagy in order to successfully discover and use autophagy-related pharmacological agents, establish their efficacy and safety in humans, and minimize their side effects. Several autophagy-related drugs (rapamycin derivates, nilotinib, bortezomib and idelalisib) have already been approved by the FDA for cancer treatment, and many more are currently being tested in clinical trials. Interestingly, autophagy-inducing natural agents (resveratrol, trehalose, fisetin, sulforaphane, lithium, and ginsenoside Rg3) have been formulated into dietary supplements and are in use to enhance neuronal health [72]. While efforts are being made to include autophagy-inducing drugs in the treatment options of many human diseases, autophagy-inducing drugs have not been approved to treat any glycogen storage diseases to this day [18]. A recent preclinical study reported that the compound 144GD11 is able to enhance glycogen-selective autophagy in in vivo and in vitro models for one glycogen storage disease: GBE deficiency [5]. The molecular target of 144DG11 is the lysosomal membrane protein LAMP1, whose interaction with the compound, similar to LAMP1 knockdown, enhances the autolysosomal degradation of glycogen and lysosomal acidification. 

Although the selective boosting of autophagy is still slightly controversial and will not cure the cause of any glycogen storage diseases, it may enable the alleviation of disease phenotypes and improve the quality of patients’ lives.

Lastly, we also identified the non-coding RNAs involved in the regulation of autophagy genes in order to increase the interest of researchers in new non-coding RNA targetsRNA-based therapeutics, such as small interfering RNAs, antisense nucleotides, splice-switching antisense oligonucleotides, messenger RNAs, etc., represent a major opportunity and new frontier for drug development. Until May 2022, sixteen RNA therapeutics have received marketing authorization from the Food and Drug Administration (FDA) in the United States of America, European Medicines Agency (EMA) in Europe, and/or the Japan Ministry of Health, Labor and Welfare [106]. Additionally, promising preclinical research has demonstrated the efficiency of small interfering RNA to silence hepatic glycogen synthase 2 (*Gys2*) expression and effectively prevent glycogen synthesis, glycogen accumulation, hepatomegaly, fibrosis, and nodule development in a mouse model of GSD III [107]. This work holds promise that in a very near future, RNA therapeutics will become relevant for the treatment of glycogen storage diseases.

## Figures and Tables

**Table 1:** Genes encoding regulatory factors related to autophagy.

**Master Genes in the Transcriptional Regulation of Autophagy**	*TFEB family, NRF2, FoxO3 family, C/EBPβ family, GATA1*
**miRNAs Involved in Autophagy Regulation**	*ULK1/2—miR-20a, miR-20b, miR-93, miR-10a, miR-106b, miR-17-5p, miR-290/295* *mTOR—miR-17-5p, miR-30A/B/C, miR-129, miR-144, miR-409-3p, miR-100* *RB1CC1—miR-20A/B, miR-224-3p* *Beclin-1—miR-17-5p, miR-124-3p, miR-216b, miR-376b* *Ambra1—miR-7, miR-23a* *UVRAG—miR-183, miR-216b, miR-351, miR-374a, miR-630,miR-1185* *ATG14—miR-152* *ATG12—miR-23a, miR-23b, miR-30, miR-214, miR-505-3p* *ATG5—miR-9a-5p, miR—142-3p, miR-181a, miR-224-3p, miR-638* *ATG7—miR-17, miR-137, miR-210, miR-520b* *RAB7—miR-138-5p* *LAMP2—miR-21, miR-207, miR-224, miR-352, miR-373-5p, miR-379, miR-487b-5p* *RAB27/LAMP3—miR-205*
**Long Non-Coding RNAs Involved in Autophagy Regulation**	*lncRNA H9, lncRNA NBR2, AD5-AlncRNA, lncRNA PTENP1, lncRNA MEG3, lncRNA ROR, lncRNA loc146880, lncRNA AC023115.3, lncRNA HOTAIRM1, lncRNA AK156230, lncRNA TGFB2, lncRNA GAS5, lncRNA HNF1A, lncRNA APF, lncRNA MALAT1, lncRNA HOTAIR, lncRNA PCGEM1, lncRNA Chast*

## Data Availability

Not applicable.
